# Prognostic significance of dysadherin expression in advanced colorectal carcinoma

**DOI:** 10.1038/sj.bjc.6600778

**Published:** 2003-03-04

**Authors:** S Aoki, T Shimamura, T Shibata, Y Nakanishi, Y Moriya, Y Sato, M Kitajima, M Sakamoto, S Hirohashi

**Affiliations:** 1 Pathology Division, National Cancer Center Research Institute, Tokyo 1-1 Tsukiji 5-Chome, Chuo-ku, 104-0045, Japan; 2 Department of Surgery, National Cancer Center Hospital, Tokyo, Japan; 3 Cancer Information and Epidemiology Division, National Cancer Center Research Institute, Tokyo, Japan; 4 Department of Surgery, Keio University School of Medicine, Tokyo, Japan; 5 Department of Pathology, Keio University School of Medicine, Tokyo, Japan

**Keywords:** dysadherin, colorectal carcinoma, immunohistochemistry, prognosis

## Abstract

A novel glycoprotein, dysadherin, has an anti-cell – cell adhesion function through downregulating E-cadherin. In this study, we investigated the expressions of dysadherin and E-cadherin in 82 patients with stage II and III colorectal carcinomas to determine the correlation between the two molecules and the clinicopathologic features of each tumour. Dysadherin was not expressed in normal colorectal epithelium. Fifty-one per cent of tumours showed dysadherin immunopositivity in over 50% of cancer cells. Thirty-eight per cent of tumours showed reduced E-cadherin immunopositivity. The increased expression of dysadherin was significantly associated with lung metastasis (*P*=0.003). The increased expression of dysadherin had a significant impact on patient survival (*P*=0.0099 and 0.0036, log-rank test for overall and recurrence-free survival rate, respectively). Furthermore, tumour with increased expression of dysadherin and reduced expression of E-cadherin showed the worst prognosis (*P*=0.0043 and 0.0028, log-rank test for overall and recurrence-free survival rate, respectively). These results suggest that increased dysadherin expression is a significant indicator of poor prognosis for patients with advanced colorectal carcinoma.

A significant number of patients with colorectal carcinoma who undergo apparently curative resection develop local recurrence or distant metastasis (Rosen *et al*, 1998). Adjuvant chemotherapy or radiation therapy is needed after resection for such biologically aggressive colorectal carcinomas. Therefore, identification of factors that accurately predict prognosis is essential.

Cell – cell interactions play pivotal roles in the maintenance and development of normal tissue (Takeichi, 1990). In cancerous tissues, disintegration of cell – cell interactions and dissemination of cancer cells lead to invasive and metastatic growth (Hirohashi, 1998). Cadherins are calcium-dependent cell adhesion molecules that play an essential role in normal growth and development via mediation of homophilic cell – cell association (Takeichi, 1990). Several studies have reported that decreased function of E-cadherin is associated with tumour progression in many cancers, and in particular E-cadherin acts as a suppressor of invasive ability (Behrens *et al*, 1989; Vleminckx *et al*, 1991; Hirohashi, 2000). Many immunohistochemical studies evaluating E-cadherin expression in tumour tissues have demonstrated that reduced expression of E-cadherin is frequently observed in cancer progression (Katagiri *et al*, 1995; Lipponen and Eskelinen, 1995; Yonemura *et al*, 1995; Tamura *et al*, 1996; Nakanishi *et al*, 1997; Umbas *et al*, 1997; Bankfalvi *et al*, 1999; Ghadimi *et al*, 1999; Gofuku *et al*, 1999; Zheng *et al*, 1999; Kase *et al*, 2000).

We previously reported a novel cancer-associated cell membrane glycoprotein, dysadherin, composed of 178 amino acids. Dysadherin is expressed in a wide variety of cancer cells, but only to a limited number of normal cells such as lymphocytes, endothelial cells, and basal cells of stratified squamous epithelium. Dysadherin inactivates E-cadherin function in a post-transcriptional manner and modulates tumour aggressiveness and metastasis (Ino *et al*, 2002). In this study we focused on the clinicopathologic significance of dysadherin and E-cadherin expression in advanced colorectal carcinoma.

## MATERIALS AND METHODS

### Materials

Eighty-two routinely processed, formalin-fixed, and paraffin wax-embedded blocks of colorectal carcinoma was obtained from the National Cancer Center Hospital. All cases were surgically resected between January 1990 and December 1990 at the National Cancer Center Hospital, and diagnosed as primary advanced colorectal carcinoma. The patients included 44 men and 38 women ranging in age from 37 to 93 years (mean, 62.6 years). The sample selection was restricted to consecutive cases diagnosed as stages II and III. All patients had undergone curative resection. None of the patients received chemotherapy or radiation therapy preoperatively. The follow-up study was complete in all patients. Recurrence after surgery was diagnosed by serum CEA level, ultrasonography, computed tomography scan, and angiography.

Thirty-five cases (42.7%) were classified as stage II, and 47 cases (57.3%) as stage III. Histologically, 52 cases were classified as well-differentiated adenocarcinoma, 27 cases as moderately differentiated adenocarcinoma, two cases as mucinous carcinoma, one case as poorly differentiated adenocarcinoma, and one case as signet-ring cell carcinoma. During the follow-up period, recurrence was observed in 10 stage II cases and 17 stage III, and the recurrence proved fatal in six stage II cases and 10 stage III cases. Tumour location, tumour differentiation, liver metastasis, lung metastasis, lymph node metastasis, lymphatic invasion, and vessel invasion were all classified according to the criteria of the Japanese Society for Cancer of the Colon and Rectum (1983).

### Immunohistochemistry

Immunohistochemical staining was performed on routinely processed, formalin-fixed, paraffin wax-embedded blocks of colorectal carcinoma tissues using an avidin–biotin peroxidase complex method. After deparaffinisation in xylene and rehydration in ethanol, the sections were heated in citrate buffer (10 mM, pH 6.0) at 120°C for 10 min for antigen retrieval. Endogenous peroxidase was blocked with 1% hydrogen peroxide in methanol for 20 min. Then, sections were incubated with anti-dysadherin antibody (M53, 1 : 500 dilution), which had been established in our laboratory and will be described elsewhere, or anti-E-cadherin antibody (1 : 200 dilution, Transduction Laboratories, US) at 4°C. The sections were washed with PBS and incubated with biotinylated anti-mouse IgG antibody and avidin–biotin complex (ABC kit, Vector Laboratories, UK) and visualised using diaminobenzidine tetrahydrochloride. The sections were counterstained with haematoxylin. As an internal positive control for dysadherin staining, positive staining of endothelial cells present in the primary tumour sections was used. As an internal positive control for E-cadherin staining, positive staining of normal colorectal epithelial cells adjacent to the tumours was used; staining was normally seen at cell – cell borders. As a negative control, normal mouse immunoglobulin of the same class was used instead of the first antibody.

### Evaluation of E-cadherin and dysadherin expression

Two observers (SA and TS), who had no previous knowledge of the clinical parameters and outcomes for each patient, independently reviewed the immunohistochemically stained sections; all discrepancies were resolved by joint review of the slides in question.

Expression. of dysadherin was considered positive if tumour cells were stained as strongly as adjacent endothelial cells. We assessed dysadherin expression as the percentage of positively stained tumour cells relative to total tumour cells. When over 50% of tumour cells were stained for dysadherin, the tumour was evaluated as ‘positive dysadherin expression’ (Dys (+)). In contrast, when fewer than 50% of tumour cells were stained for dysadherin, the tumour was evaluated as ‘negative dysadherin expression’ (Dys (−)).

Expression of E-cadherin was considered positive if tumour cells were stained as strongly as normal epithelial cells adjacent to the tumour, whereas those that stained more weakly than normal epithelial cells were considered negative. We also assessed E-cadherin expression as the percentage of positively stained tumour cells relative to total tumour cells. When over 80% of tumour cells were positive, the tumour was evaluated as ‘preserved E-cadherin expression’ (E-cad (+)). Whereas the tumour was evaluated as ‘reduced E-cadherin expression’ (E-cad (−)) when fewer than 80% of tumour cells were positive.

### Statistical analysis

The relation of clinicopathologic characteristics to the number of immunopositive tumour cells was analysed using the *χ*^2^ test and ANOVA test where appropriate. Deaths from causes other than colorectal carcinoma were treated as censored cases. Recurrence-free survival and overall survival were measured from the date of surgery to the date that the recurrence was confirmed and the end of follow-up or death, respectively. Overall survival curves were determined by using the Kaplan – Meier method and were analysed with the log-rank test. Multivariate survival analysis was performed with the Cox proportional hazards regression model. Differences at *P*<0.05 were regarded as statistically significant.

## RESULTS

### Expression of dysadherin

**Figure 1:**
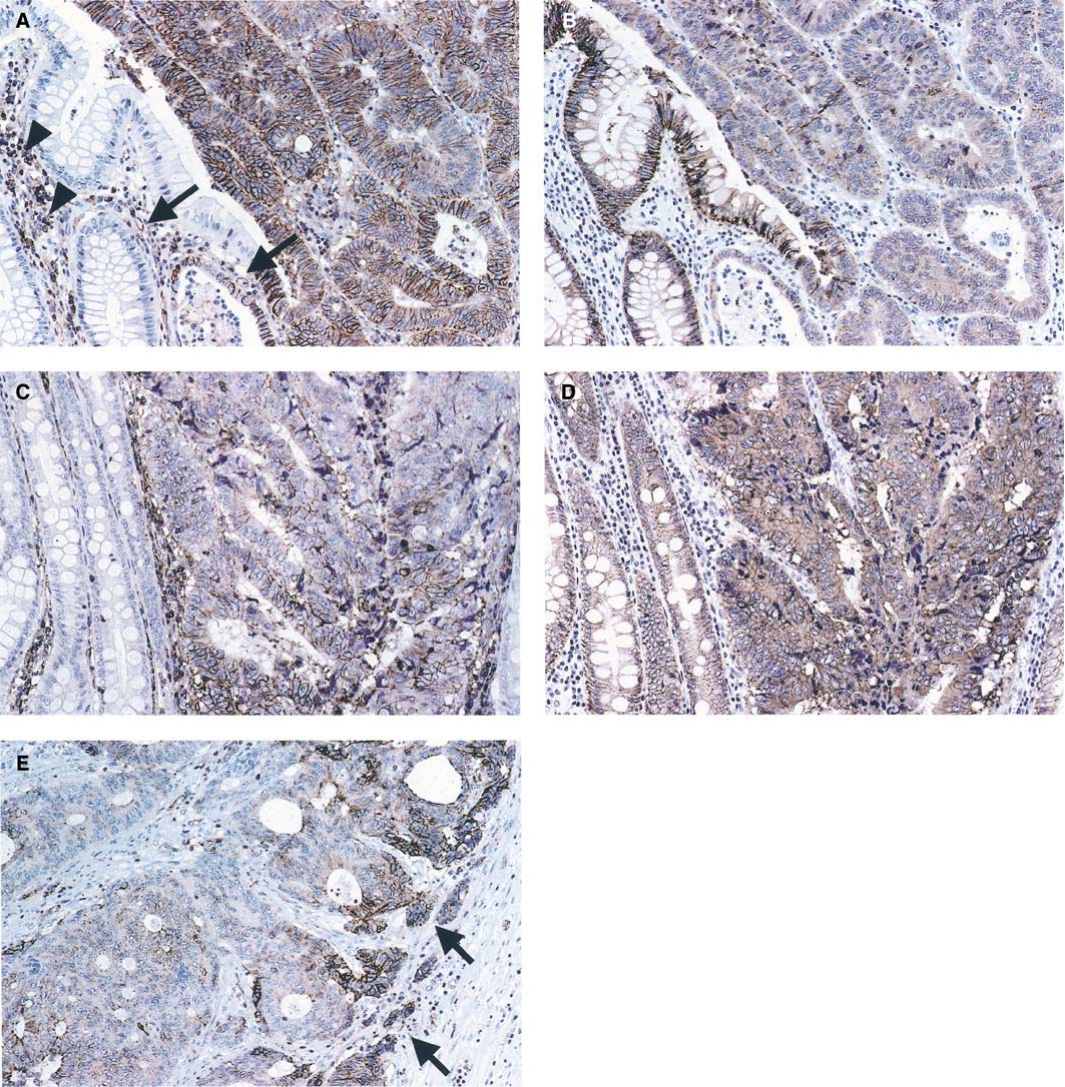
Dysadherin and E-cadherin expression in colorectal cancers demonstrated by immunohistochemistry. (**A**)–(**D**) are the adjacent sections of the same cases. (**A**) Dysadherin expression was observed in lymphocytes (arrowheads) and endothelial cells (arrows). Membranous dysadherin staining was localised at intercellular borders of cancer cells and was heterogeneous in tumour nests. No immunoreactivity was seen in normal colorectal epithelial cells (magnification × 200). (**B**) E-cadherin expression was observed at the cell–cell borders in normal epithelial cells, and was reduced in the majority of cancer cells in this section (magnification × 200). (**C**) Tumour cells were positively stained for dysadherin (magnification × 200). (**D**) E-cadherin expression was not reduced in tumour cells where dysadherin was expressed (magnification × 200). (**E**) Preferential dysadherin expression was observed in infiltrative tumour cells (arrow) in some cases (magnification × 200).

Dysadherin-positive staining was observed at the membranes of cancer cells, lymphocytes, and endothelial cells as previously reported (Ino *et al*, 2002). Membranous dysadherin staining was localised at intercellular borders of cancer cells and was heterogeneous in tumour nests. No immunoreactivity was seen in normal colorectal epithelial cells (Figure 1A, C

). Preferential expression was observed in infiltrative tumour cells in some cases (Figure 1E). The mean percentage of tumour cells that stained positively for dysadherin was 52.0±32.3% (mean±standard deviation (s.d.); median, 45%). Therefore, we set the cut-off value for dysadherin immunopositivity at 50%, which made it simple for observers to categorise each case according to dysadherin expression. Thirty-nine per cent (32 out of 82) of cases demonstrated immunopositivity in over 50% of cancer cells.

The correlations between dysadherin expression and various clinicopathological factors were analysed. Increased dysadherin expression was significantly correlated with lung metastasis (*P*=0.003). There was no significant correlation between the increased expression of dysadherin and other clinicopathological factors such as age, gender, tumour location, maximum diameter of the tumour, macroscopic type, TNM stage, tumour differentiation, liver metastasis, lymphatic invasion, and vessel invasion (Table 1

**Table 1:** Correlation between expression of E-cadherin and dysadherin and clinicopathological characteristics in patients with advanced colorectal carcinoma (stages II and III)

	**E-cadherin**	**Dysadherin**				
**Variables**	**Preserved**	**Reduced**	***P*-value**	**Fewer than 50%**	**Over 50%**	***P*-value**
Age (mean±s.d.)(years)	62.3±10.9	62.0±10.8	0.784	62.2±11.6	63.0±10.4	0.746
*Gender*						
Male	31	13		24	20	
Female	20	18	0.097	26	12	0.199
*Tumour location*						
Colon	24	16		27	13	
Rectum	27	15	0.689	23	19	0.237
Maximum diameter of the tumour						
Mean±s.d. (cm)	5.21±2.23	5.47±2.31	0.767	5.67±2.15	5.08±2.34	0.247
*Macroscopic type*^a^						
1	2	4		3	3	
2	43	23		41	25	
3	5	4		5	4	
4	1	0	0.369	1	0	0.779
*TNM stage*						
II	22	13		23	12	
III	29	18	0.915	27	20	0.448
*Tumour Differentiation*^b^						
Well	32	19		33	18	
Mod, por, sig, muci	19	12	0.895	17	14	0.376
*Liver metastasis*						
Positive	5	8		7	6	
Negative	46	23	0.054	43	26	0.566
*Lung metastasis*						
Positive	6	2		1	7	
Negative	45	29	0.431	49	25	0.003
*Lymphatic invasion*						
Positive	23	21		37	26	
Negative	28	10	0.046	13	6	0.448
*Vessel invasion*						
Positive	14	6		11	9	
Negative	37	25	0.408	39	23	0.529

a Corresponding to Borrman's classification: 1=polypoid; 2=ulcerated circumscribed; 3=ulcerated infiltrative; 4=diffusely infiltrative.

b Well=well-differentiated adenocarcinoma; mod=moderately differentiated adenocarcinoma; poor=poorly differentiated adenocarcinoma; sig=signetring cell carcinoma; muci=mucinous carcinoma.

).

### Expression of E-cadherin

E-cadherin-positive staining was observed at the cell – cell borders in normal colorectal epithelial cells and in the majority of cancer cells (Figure 1B, D). Preserved membranous E-cadherin staining, with an intensity equal to that of the adjacent normal epithelium, was present in 62.2% (51 out of 82) of cases (Figure 1D). E-cadherin immunoreactivity was reduced in 37.8% (31 out of 82) of cases (Figure 1B).

The reduced expression of E-cadherin was correlated only with lymphatic invasion (*P*=0.046). There was no significant correlation between the reduced expression of E-cadherin and age, gender, tumour location, maximum diameter of the tumour, macroscopic type, TNM stage, tumour differentiation, liver metastasis, lung metastasis, and vessel invasion (Table 1).

In this analysis, dysadherin expression (cut-off, 50%) was not correlated with E-cadherin expression (*P*=0.3744) (Table 2

**Table 2:** Cases classified by dysadherin expression and E-cadherin expression

	**Dysadherin expression**	
**E-cadherin expression**	**Fewer than 50%**	**Over 50%**
Preserved	33	18
Reduced	17	14

*P*=0.3744.

).

### Prognostic value of dysadherin and E-cadherin expression

**Figure 2:**
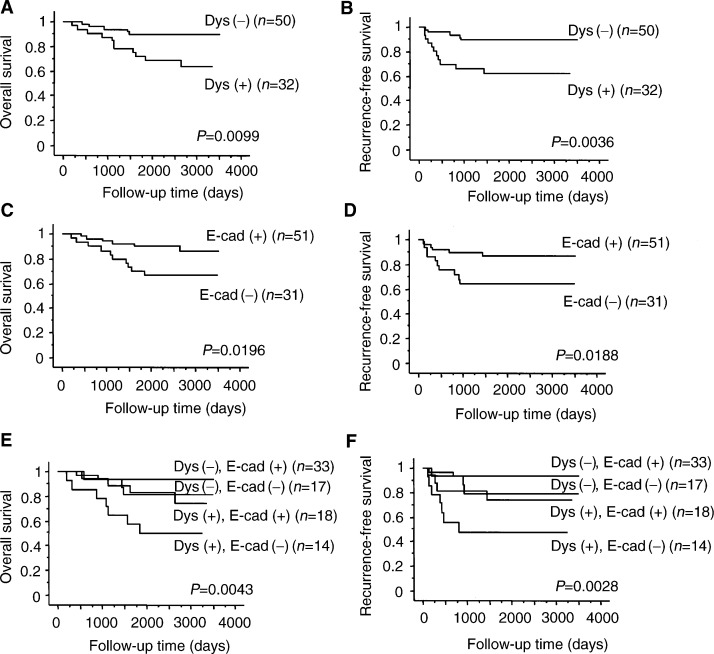
Overall survival (**A**) and recurrence-free survival (**B**) of patients in relation to dysadherin expression (log-rank test for trend: *P*=0.010, 0.004, respectively). Overall survival (**C**) and recurrence-free survival (**D**) of patients in relation to E-cadherin expression (log-rank test for trend: *P*=0.020, 0.020, respectively). Overall survival (**E**) and recurrence-free survival (**F**) of patients in relation to the combination of dysadherin and E-cadherin expression (log-rank test for trend: *P*=0.004, 0.003, respectively). Dys (+), dysadherin expression in over 50% of tumour cells; Dys (−), dysadherin expression in fewer than 50% of tumour cells; E-cad (+), E-cadherin expression in over 80% of tumour cells; E-cad (−), E-cadherin expression in fewer than 80% of tumour cells.

Patients with dysadherin immunopositivity in over 50% of tumour cells survived significantly shorter than those with dysadherin immunopositivity in fewer than 50% of tumour cells (*P*=0.0099. and 0.0036, log-rank test for overall and recurrence-free survival rates, respectively; Figure 2 A, B

)

Patients with reduced E-cadherin immunopositivity survived significantly shorter than those with preserved E-cadherin immunopositivity (*P*=0.0196 and 0.0188, log-rank test for overall and recurrence-free survival rates, respectively; Figure 2C, D).

Patients with combined dysadherin immunopositivity in over 50% of tumour cells and reduced E-cadherin immunopositivity survived significantly shorter than those with other combinations of dysadherin and E-cadherin immunopositivity (*P*=0.0043 and 0.0028, log-rank test for overall and recurrence-free survival rates, respectively; Figure 2E, F).

Multivariate analysis using Cox's proportional hazards model revealed that dysadherin immunopositivity and E-cadherin immunopositivity were independent and significant prognostic factors when adjusted for age, gender, maximum tumour diameter, tumour differentiation, lymph node metastasis, lymphatic invasion, and vessel invasion (Table 3

**Table 3:** Multivariate Cox's proportional hazard analysis

	**Overall survival**			**Recurrence-free survival**		
**Prognostic factors**	**HR**	**95% CI**	***P*-value**	**HR**	**95% CI**	***P*-value**
Dysadherin	3.497	1.032–11.851	0.0444	2.522	1.064–5.977	0.0356
E-cadherin	3.588	1.075–11.982	0.0378	2.393	1.013–5.653	0.0466
Gender	0.957	0.957–0.264	0.9468	0.91	0.363–2.282	0.8412
Age	3.751	0.533–26.372	0.184	1.339	0.372–4.822	0.6554
Maximum diameter of the tumour	1.729	0.379–7.886	0.4793	1.164	0.408–3.324	0.7765
Tumour differentiation (well *vs* others)	2.116	0.612–7.323	0.2366	1.2	0.479–3.009	0.6972
Lymphatic invasion	1.143	0.247–5.281	0.864	1.406	0.450–4.388	0.5578
Vessel invasion	1.601	0.379–6.767	0.5219	1.262	0.449–3.546	0.6592
Lymph node metastasis	1.213	0.367–4.013	0.7516	1.538	0.639–3.702	0.3369

HR=hazard ratio; CI=confidence interval.

).

## DISCUSSION

We reported previously that dysadherin has an anti-cell – cell adhesion function and downregulates E-cadherin, on the basis of the observation that introduction of dysadherin induced decreased the protein expression and function of E-cadherin without affecting its transcription (Ino *et al*, 2002). In various cancerous tissues, a reduced expression of E-cadherin has frequently been observed in cancer progression (Katagiri *et al*, 1995; Lipponen and Eskelinen, 1995; Yonemura *et al*, 1995; Tamura *et al*, 1996; Nakanishi *et al*, 1997; Umbas *et al*, 1997; Bankfalvi *et al*, 1999; Ghadimi *et al*, 1999; Zheng *et al*, 1999; Kase *et al*, 2000; Gofuku *et al*, 1999). The aim of this study was to verify immunohistochemically the hypothesis that dysadherin, which suppresses the E-cadherin system, might play a significant role in the aggressiveness of colorectal carcinoma.

As in our previous study (Ino *et al*, 2002), we confirmed that dysadherin was expressed heterogeneously in tumour nests, in that it was expressed in dissociating cells or infiltrative tumour nests and also in parts of well-differentiated tumour nests. As reported, dysadherin was not detected in normal colorectal epithelium, but was expressed significantly in endothelial cells. Use of these cells as internal controls allowed easy assessment of dysadherin expression by cancer cells.

The metastatic process involves a complex series of events that must include changes in the status of adhesion molecules of the tumour cells. E-cadherin, which is responsible for calcium-dependent homophilic interaction between epithelial cells, has been shown to be downregulated in several tumours including colorectal carcinoma (Ghadimi *et al*, 1999; Gofuku *et al*, 1999). Several mechanisms have been proposed for the inactivation of the cadherin/catenin complex in tumour cells, such as mutations in the genes for E-cadherin, α-catenin, and β-catenin (Hirano *et al*, 1992; Oda *et al*, 1994; Oyama *et al*, 1994; Machado *et al*, 1999), hypermethylation around the promoter region of the E-cadherin gene (Yoshiura *et al*, 1995), and aberrant tyrosine phosphorylation of β-catenin (Behrens *et al*, 1993). In this study, reduced E-cadherin expression was observed in 37.8% (31 out of 82) of tumours, which was less than reported previously (Dorudi *et al*, 1995; Mohri, 1997; Ikeguchi *et al*, 2000). This discrepancy may be a result of the different evaluation system for immunopositivity or of the different cases examined; we restricted sample selection to consecutive cases diagnosed as stages II and III. However, as in previous studies (Dorudi *et al*, 1995; Mohri, 1997; Ikeguchi *et al*, 2000), the present immunohistochemical data demonstrate that reduced E-cadherin expression is significantly correlated with poor survival.

We demonstrated previously that dysadherin had some role in post-transcriptional downregulation of E-cadherin (Ino *et al*, 2002). In this immunohistochemical study, although dysadherin was detected in part of some tumours, where E-cadherin expression was decreased, positive dysadherin expression was not correlated with reduced E-cadherin expression. This result suggests that mechanisms may exist for the reduction of E-cadherin expression other than that related to dysadherin expression. Our immunohistochemical data also demonstrate that increased dysadherin expression is significantly correlated with poor survival. Furthermore, tumours with both increased expression of dysadherin and reduced expression of E-cadherin showed the worst prognosis, and tumours with the opposite combination of the two molecules showed the best prognosis. These results suggest that dysadherin expression might be an excellent indicator of the patients with advanced colorectal carcinoma and the combined immunohistochemical analysis of dysadherin and E-cadherin expression could give us further prognostic information. In the current study we categorised patients simply into two groups setting the cut-off value for dysadherin immunopositivity at 50%, on the ground that the mean percentage of dysadherin-positive tumour cells was 52.0±32.3% (mean±s.d.; median, 45%). We also confirmed the prognostic importance of dysadherin positivity by classifying dysadherin expression into four categories (0–10, 10–30, 30–50, more than 50% positivity) (data not shown).

Although curative resection of advanced colorectal carcinoma without synchronous distant metastasis (ie stage II or III) is feasible, a significant number of patients develop local recurrence or distant metastasis heterochronously (Newland *et al*, 1995; Rosen *et al*, 1998). Liver metastasis is an important prognostic determinant for the clinical prognosis of colorectal carcinoma (Doci *et al*, 1991; Wanebo *et al*, 1996; Rees *et al*, 1997). Therefore, a prognostic indicator correlated with liver metastasis and that can be assessed at the time of resection of the primary tumour is required to aid in the selection of patients for adjuvant therapy. Tumour suppressor genes such as nm23 (Yamaguchi *et al*, 1993), DCC (Saito *et al*, 1999) and Smad4 (Miyaki *et al*, 1999) have been reported to play a role in liver metastasis. As for adhesion molecules, reduction of the E-cadherin–catenin system (Hirohashi, 1998) and expression of variant CD44 (Wielenga *et al*, 1993) have been studied as biological markers of liver metastasis. In the previous study (Ino *et al*, 2002), we demonstrated that dysadherin promoted the metastatic activity by *in vivo* metastasis assay. In this study positive dysadherin expression was observed in six of &1QJ;13 cases, which developed liver metastasis postoperatively. Considering that various mechanisms may be involved in liver metastasis and stage IV colorectal carcinoma, including synchronous liver metastasis, were out of subject in our current study, further analysis will be required to examine the relation between dysadherin expression and liver metastasis.

Interestingly, positive dysadherin expression was observed in seven of eight cases which developed lung metastasis heterochronously. In three out of those eight cases, the metastatic lesions were surgically resected. Immunohistochemically, dysadherin was expressed heterogeneously in all three lung metastatic lesions as much as in the corresponding primary tumours (data not shown). Although the process of lung metastasis of colorectal carcinoma is still unclear, dysadherin might present a predictive information at the time of the resection of primary lesion.

In conclusion, we have demonstrated a significant correlation between the expression of dysadherin and the prognosis of patients with advanced colorectal carcinoma. Univariate and multivariate analyses revealed that the expression of dysadherin in tumour cells might be an excellent and independent prognostic indicator for patients with advanced colorectal carcinoma.
